# B7h-expressing dendritic cells and plasma B cells mediate distinct outcomes of ICOS costimulation in T cell-dependent antibody responses

**DOI:** 10.1186/1471-2172-13-29

**Published:** 2012-06-11

**Authors:** Kevin Larimore, Linda Liang, Sonia Bakkour, William C Sha

**Affiliations:** 1 Immunology Division, Department of Molecular and Cell Biology, University of California, Berkeley, CA, 94720-3200, USA

**Keywords:** ICOS, B7h, Costimulation, Antibody, Germinal center, Plasma cell, Dendritic cell

## Abstract

**Background:**

The ICOS-B7h costimulatory receptor-ligand pair is required for germinal center formation, the production of isotype-switched antibodies, and antibody affinity maturation in response to T cell-dependent antigens. However, the potentially distinct roles of regulated B7h expression on B cells and dendritic cells in T cell-dependent antibody responses have not been defined.

**Results:**

We generated transgenic mice with lineage-restricted B7h expression to assess the cell-type specific roles of B7h expression on B cells and dendritic cells in regulating T cell-dependent antibody responses. Our results show that endogenous B7h expression is reduced on B cells after activation in vitro and is also reduced in vivo on antibody-secreting plasma B cells in comparison to both naïve and germinal center B cells from which they are derived. Increasing the level of B7h expression on activated and plasma B cells in B-B7hTg mice led to an increase in the number of antibody-secreting plasma cells generated after immunization and a corresponding increase in the concentration of antigen-specific high affinity serum IgG antibodies of all isotypes, without affecting the number of responding germinal center B cells. In contrast, ICOS costimulation mediated by dendritic cells in DC-B7hTg mice contributed to germinal center formation and selectively increased IgG2a production without affecting the overall magnitude of antibody responses.

**Conclusions:**

Using transgenic mice with lineage-restricted B7h expression, we have revealed distinct roles of ICOS costimulation mediated by dendritic cells and B cells in the regulation of T cell-dependent antibody responses.

## Background

ICOS is an inducible costimulatory receptor expressed on activated T cells that is a member of the CD28-B7 family of costimulatory molecules [1-4]. ICOS binds to the ligand B7h [5] (also known as LICOS [6], ICOSL [7], GL50 [8], B7RP-1 [9], and B7-H2 [10]), expressed constitutively on the cell surface of resting B cells and dendritic cells (DCs) [5,9,11,12], both of which can regulate T cell-dependent antibody responses. Studies of B7h^−/−^and ICOS^−/−^ mice have demonstrated the requirement of the ICOS-B7h receptor-ligand pair in germinal center formation, class switched antibody production and antibody affinity maturation [7,13-15], but the potentially distinct roles of ICOS costimulation mediated by B7h-expressing B cells and DCs in the regulation of antibody responses have not been well defined.

ICOS signaling can promote IL-4 production, leading to Th2 polarization of differentiating CD4+ T cells [16,17], but can also enhance production of a variety of cytokines in other Th subsets that have already differentiated, including augmentation of IFN-γ production in Th1 cells [18-20]. Thus, ICOS signaling in T cells can have different effects on immune responses depending upon the cellular context of ICOS-B7h interactions. In T cell-dependent antibody responses, ICOS-expressing activated CD4+ T cells can make contact with B7h-expressing antigen presenting cells in several distinct contexts, with the potential for regulation of different aspects of the response through ICOS signaling during each interaction. DCs can contact recently-activated CD4+ ICOS+ T cells in T cell zones [21], and DCs in germinal centers can interact with ICOS+ Tfh cells [22]. Recently activated B cells interact with cognate activated CD4+ T cells at the border of the T cell and B cell zones in lymph nodes and spleen prior to germinal center and plasma cell formation [22], germinal center B cells interact with ICOS+ follicular helper T (Tfh) cells [23], and antibody-secreting plasma B cells interact with activated Th cells in periarteriolar lymphoid sheaths [24]. Because the outcome of ICOS signaling in CD4+ T cells depends upon the differentiation and programming of T cells in each context [25], these interactions represent potentially distinct points of control for antibody responses, where modulation of ICOS signaling by regulated B7h expression on antigen presenting cells could have different effects. 

Previous in vitro studies have shown that down-regulation of B7h expression on B cells after activation can restrict ICOS costimulation in cognate CD4+ T cells, suggesting that regulation of B7h levels on activated B cells in vivo could be a control mechanism in T cell-dependent antibody responses. B7h expression on activated B cells is transcriptionally extinguished by exposure to antigen and IL-4 [26], and is also limited by rapid ectodomain shedding induced either by binding to ICOS or by antigen receptor signaling [27]. Conversely, B7h expression can be restored by the reactivation of transcription induced by CD40 signaling [26], and enhanced by the inhibition of ectodomain shedding induced by TLR7/8 and TLR9 signaling [27]. Thus, the level of B7h expression on activated B cells reflects the integration of multiple critical B cell signaling pathways. 

To investigate the cell type-specific functions of regulated B7h expression on B cells and DCs in vivo, we generated transgenic mice with lineage-restricted B7h expression. Our results show that ICOS costimulation mediated by DCs contributes to germinal center formation in response to T cell-dependent antigens and leads to a selective increase in antibody class switching to the IgG2a isotype, without affecting the overall magnitude of antibody responses. In contrast, increased expression of B7h on B cells, but not on dendritic cells, markedly enhances both the number of plasma cells secreting antigen-specific high affinity class-switched antibodies and the serum concentration of such antibodies, without affecting the number of germinal center B cells or antibody isotype usage. Thus, we have defined distinct roles of ICOS costimulation mediated by DCs and plasma B cells in T cell-dependent antibody responses.

## Results

### DC-B7hTg mice overexpress B7h on CD11c + dendritic cells

**Figure 1:**
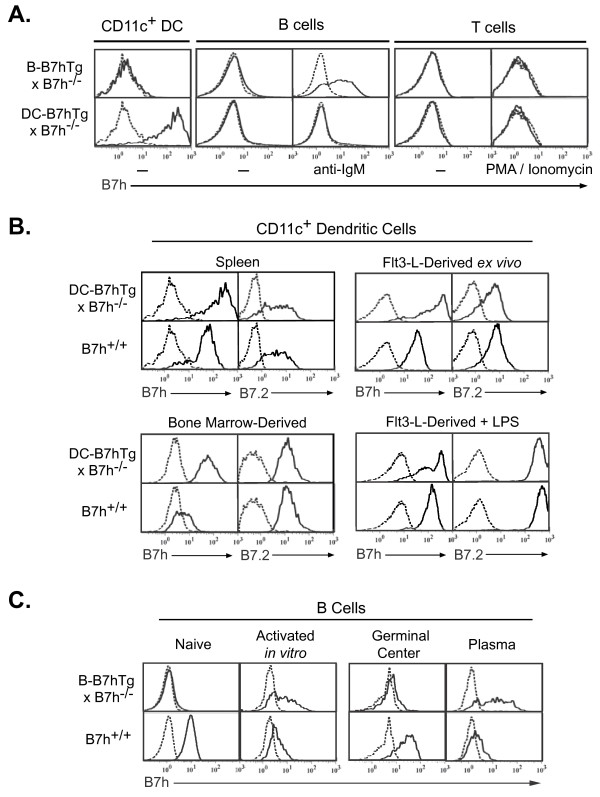
**Lineage-specific B7h transgene expression on B cells from B-B7hTg mice after activation and on dendritic cells from DC-B7hTg mice.** B7h transgene expression in B-B7hTg and DC-B7hTg mice was assessed by flow cytometry on the B7h^−/−^ background to avoid contribution from endogenous B7h. B7h staining is shown (solid line) after gating on the indicated cell subsets, with background staining of B7h^−/−^ controls (dashed line). (**A**) B7h transgene expression on resting splenocytes was assessed using CD11c, B220, and Thy1 as markers for DCs, B cells and T cells, respectively. To assess transgene expression on activated B and T cells, purified resting splenic B and T cells were activated for 16 hours with anti-IgM F(ab’)_2_ or PMA and ionomycin, respectively. (**B**) B7h and B7.2 expression were examined on splenic CD11c+ dendritic cells from naive mice, purified CD11c+ dendritic cells derived from bone marrow, and splenic CD11c+ cells purified from mice injected with Flt3-ligand-expressing B16 cells, either directly ex vivo or after 16 hours of treatment with LPS. For B7.2 analysis, dashed lines represent the background staining of a non-specific isotype control antibody. (**C**) B7h staining of purified resting splenic B cells, gated B220^+^, was assessed before and after 3 days of stimulation with anti-IgM F(ab’)_2_**.** B7h staining of B220^+^IgD^neg-^GL7^+^Fas^+^ germinal center B cells and IgD^neg-^syndecan-1^+^B220^lo^ plasma B cells was assessed at day 7 in mice immunized with NP-CGG in alum.

To investigate the in vivo roles of B7h expression on dendritic cells and B cells, both of which can regulate T cell-dependent antibody responses, we generated transgenic mice with lineage-restricted B7h expression. We targeted the expression of B7h to DCs using a transgenic expression vector driven by the CD11c promoter [28]. The DC-B7hTg line was selected from founder mice based on overexpression of B7h on CD11c + cells, and dendritic cell-specific transgene expression was confirmed by analyzing DC-B7hTg mice on the B7h^−/−^ background to avoid any contribution from endogenous B7h (Figure 1A). Flow cytometric analysis revealed that B7h transgene expression in DC-B7hTg mice was restricted to CD11c+ DCs, and was not expressed on either resting or in vitro-activated B or T cells. Expression of the DC-B7h transgene was significantly higher than endogenous B7h on both resting and activated DCs (Figure 1B), indicating that the DC-B7hTg line could be used to study the potential regulatory role of enhanced B7h expression on dendritic cells in vivo. 

### The B-B7h transgene drives B7h expression on activated B cells

Expression of B7h was also targeted to B cells using a transgenic expression vector driven by an immunoglobulin heavy chain VH promoter and the intronic Eμ enhancer [29]. Since BCR cross-linking induces substantial down-regulation of endogenous B7h on B cells [26], we selected the B-B7hTg line from founder mice based on enhanced B7h expression on anti-IgM-activated B cells. To confirm B cell-specific transgene expression, we analyzed B7h expression on splenocytes from B-B7hTg mice on the B7h^−/−^ background to avoid any contribution from endogenous B7h (Figure 1A). Although transgene expression was restricted to B220+ cells, and was not expressed on either dendritic cells or resting or activated T cells, transgene expression on naïve B cells was very low. Significant transgenic B7h expression, approaching the level of endogenous B7h on naïve wild-type B cells, required extended in vitro B cell activation with anti-IgM antibodies (Figure 1A: 16 hr stimulation; Figure 1C: 3 day stimulation). Thus, the B7h transgene in B-B7hTg mice was restricted to B cells and was induced after activation.

The essentially wild-type levels of B7h on naive B-B7hTg B cells on the B7h^+/+^ background allowed us to focus specifically on testing the in vivo significance of endogenous B7h down-regulation on activated B cells. A prior study attempting to analyze the reciprocal question of the in vivo significance of B7.2 induction on activated B cells employed the analogous approach of generating transgenic mice expressing a B cell-specific B7.2 transgene [30]. Interpretation of antibody responses in these mice, however, was precluded by a severe reduction in the number of resting naïve B cells that resulted from the inappropriate expression of B7.2 on resting B cells. In contrast, both B-B7hTg mice, with restricted B7h transgene expression on activated B cells, and DC-B7hTg mice, with restricted B7h transgene expression on CD11c+ DCs, exhibited normal numbers of B220+, CD4+, CD8+, and CD11c + splenocytes (Additional file 1: Figure S1), consistent with phenotypically normal resting immune systems.

### Endogenous B7h expression is limited on plasma B cells

Although it was known that B cell receptor and IL-4R activation of B cells led to extinguishment of B7h expression in vitro [26], the corresponding in vivo expression of B7h on activated germinal center and plasma B cell subsets was not known at the time we began our studies. In wild-type C57Bl6 mice immunized with the T cell-dependent antigen NP-CGG (4-Hydroxy-3-nitrophenylacetyl hapten conjugated to chicken gamma globulin)in alum, B7h was highly expressed on B220^+^IgD^+^ naïve B cells and B220^+^IgD^neg^GL7^+^Fas^+^ germinal center B cells, but the expression level was dramatically reduced on IgD^neg^Syndecan-1^+^B220^lo^ plasma B cells (Figure 1C). In contrast, transgene-driven B7h expression in immunized B-B7hTg mice on the B7h^−/−^ background was barely detectable on naïve and germinal center B cells, but appeared to be selectively induced on responding plasma B cells.

### The B-B7h transgene drives overexpression of B7h on activated and plasma B cells in the presence of endogenous B7h

**Figure 2:**
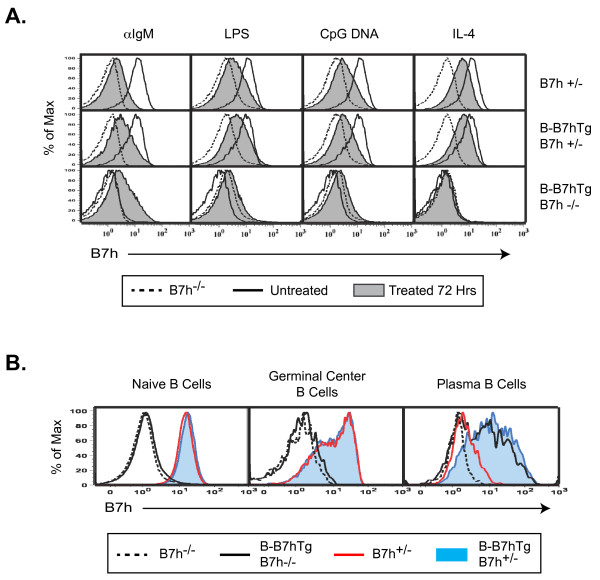
**The B-B7h transgene is overexpressed on activated and plasma B cells in the presence of endogenous B7h.** (**A**) Purified B cells from B7h^+/−^, B7h^−/−^, and B-B7hTg mice on the B7h^+/−^ and B7h^−/−^ backgrounds were cultured for 72 hours in the presence of activating stimuli, and cell surface B7h levels were measured by FACS. Dashed lines represent staining of B7h on B7h^−/−^ cells cultured in the presence of stimuli; solid lines represent cells cultured without stimuli, and shaded histograms represent cells cultured with stimuli, from mice of the genotypes shown on the right. (**B**) Seven days after immunization with NP-CGG, splenocytes from mice with the genotypes indicated in the legend were analyzed by FACS for cell-surface B7h levels and lineage markers. Naïve B cells were gated B220^+^IgD^+^, germinal center B cells were gated B220^+^IgD^neg^GL7^+^CD95^+^, and plasma cells were gated IgD^neg^B220^lo^CD138^+^.

Having observed induction of B7h expression on B cells from B-B7hTg mice on the B7h^−/−^ background after in vitro activation or in vivo differentiation to the plasma cell lineage, and reduction of B7h expression on WT B cells under the same conditions, we sought to determine whether the presence of the B-B7h transgene would lead to overexpression of B7h on activated and plasma B cells in the presence of endogenous B7h. To assess B7h expression on activated B cells, we purified B cells from naïve B7h^−/−^, B7h^+/−^, and B-B7hTg mice on the B7h^−/−^ and B7h^+/−^ backgrounds, cultured the cells for 72 hours in the presence of B cell stimuli, and measured cell-surface B7h expression by FACS (Figure 2A). Endogenous B7h expression was high on untreated B cells and was reduced after culture with an activating anti-IgM antibody F(ab’)_2_, LPS, IL-4, or CpG oligonucleotide, consistent with previous observations [26]. Conversely, on the B7h^−/−^ background the B-B7h transgene was not expressed on untreated cells, was strongly induced by IgM cross-linking, and was induced at a low level by culture with LPS or CpG DNA, but not IL-4. B cells from B-B7hTg mice on the B7h^+/−^ background expressed B7h on the cell surface at levels equivalent to non-transgenic controls directly ex vivo, and activating stimuli led to down-regulation of B7h expression after culture in vitro. However, the level of cell-surface B7h after IgM cross-linking was markedly higher on cells from B-B7hTg mice on the B7h^+/−^ background compared with anti-IgM-treated B cells from B7h^+/−^ mice, suggesting that induction of transgenic B7h expression led to overexpression of B7h on B cells after BCR-mediated activation, when endogenous B7h expression was limited. 

To determine whether the B-B7h transgene led to overexpression of B7h on plasma B cells in the presence of endogenous B7h in vivo, we isolated splenocytes from B7h^−/−^ and B7h^+/−^ mice, as well as B-B7hTg mice on the B7h^−/−^ and B7h^+/−^ backgrounds, 7 days after immunization with NP-CGG, and analyzed B7h expression on B cell subsets by FACS (Figure 2B). On both B220^+^IgD^+^ naïve B cells and B220^+^IgD^neg^GL7^+^CD95^+^ germinal center B cells, B7h expression was equivalently high on cells from B7h^+/−^ mice and B-B7hTg mice on the B7h^+/−^ background, and barely detectable on cells from B-B7hTg mice on the B7h^−/−^ background, indicating that the B-B7h transgene did not induce B7h expression in these subsets. In contrast, IgD^neg^B220^lo^CD138^+^ plasma B cells from B7h^+/−^ mice expressed low levels of cell surface B7h, while B-B7hTg mice on both the B7h^+/−^ and B7h^−/−^ backgrounds expressed high levels of B7h, confirming that the B-B7h transgene was induced on plasma B cells, leading to overexpression in the presence of endogenous B7h. These results suggest that ICOS costimulation might normally be limited by down-regulation of endogenous B7h expression on recently-activated B cells and differentiated plasma B cells, and the observed induction of B7h on activated and plasma B cells in B-B7hTg mice could be used to test the functional significance of regulated B7h expression on wild-type B cells in vivo.

### B7h expression on B cells regulates the magnitude of T cell-dependent antibody responses

**Figure 3:**
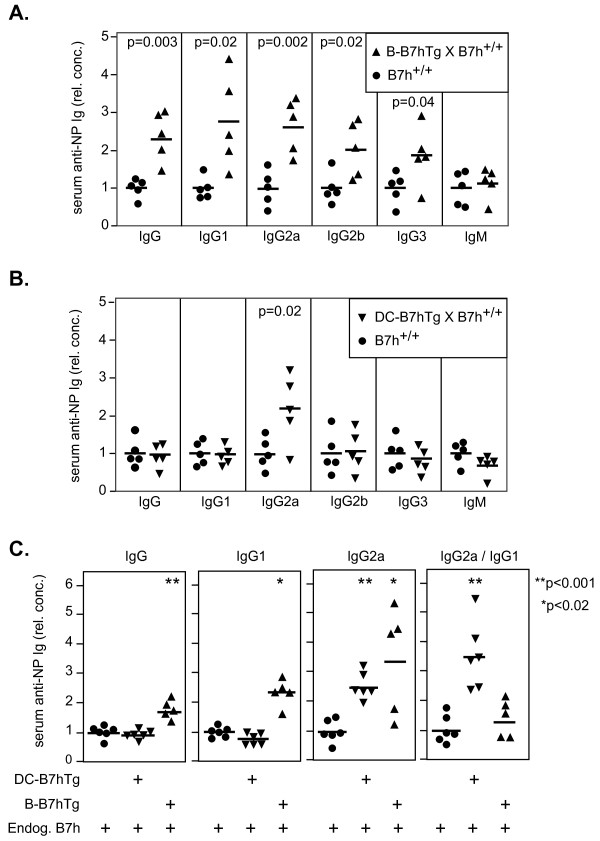
**ICOS costimulation mediated by plasma B cells and DCs selectively regulate the magnitude and isotype usage of antibody responses.** Transgenic mice on the B7h^+/+^ background and non-transgenic littermate controls were immunized with NP-CGG in alum, and serum antibody responses were analyzed by ELISA. Relative isotype-specific anti-NP serum antibody concentrations at day 7 are shown for B-B7hTg (**A**) and DC-B7hTg (**B**) mice, compared with wild-type controls. (**C**) The relative serum concentrations of anti-NP IgG, IgG1, IgG2a, and the IgG2a/IgG1 ratio are shown at day 14 for transgenic and wild-type mice. Each symbol represents an individual immunized mouse and the mean of each group is indicated by a solid bar. The data were normalized such that control groups have mean value of 1. Asterisks and p values are shown where statistical significance (Student’s *t* test, p < 0.05) was detected between transgenic and control groups. The data are representative of at least 3 independent experiments.

B-B7hTg mice and non-transgenic littermates on the B7h^+/+^ background were immunized with the T cell-dependent antigen NP-CGG in alum, and anti-NP serum antibody concentrations were measured after 7 days by ELISA (Figure 3A). Total anti-NP IgG, but not anti-NP IgM responses were significantly increased in B-B7hTg mice over non-transgenic littermate controls. The increase in total anti-NP IgG responses of B-B7hTg mice was reflected in all IgG isotypes examined, suggesting that the level of B7h expression on responding plasma B cells regulated the overall magnitude of the IgG response through ICOS costimulation. Investigation of serum antibody responses on day 14 after immunization (Figure 3C) confirmed the increase in antigen-specific antibody concentration in B-B7hTg mice over controls, while the IgG2a/IgG1 isotype ratio was equivalent to that seen in wild-type mice, suggesting that plasma B cell-mediated ICOS signaling did not affect antibody isotype usage.

**Figure 4:**
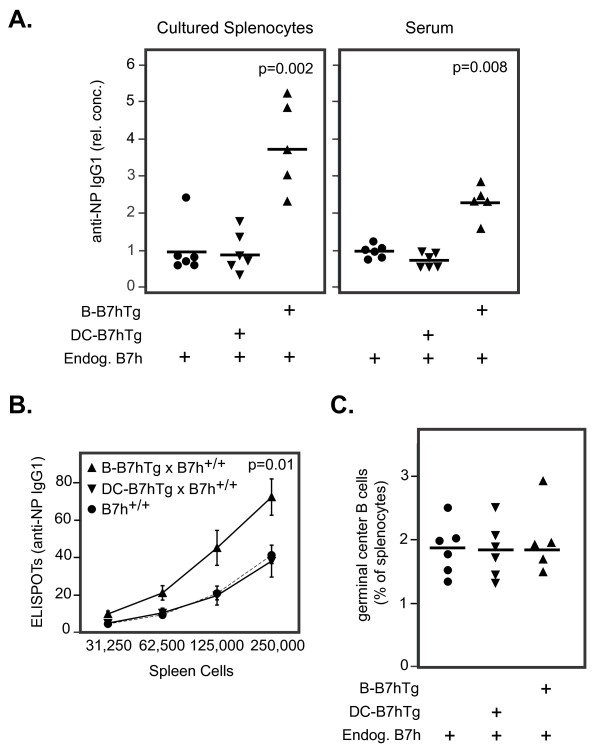
**The level of B7h on plasma B cells regulates the number of antibody secreting cells generated by immunization.** Transgenic and control mice on the B7h^+/+^ background were immunized with NP-CGG and analyzed after 14 days. (**A**) The relative concentration of anti-NP IgG1 antibodies in supernatants from overnight splenocyte cultures and in serum were measured by ELISA. Each symbol represents one mouse and bars indicate the mean of each group. The data were normalized such that the mean of the control group had a value of 1. P values are shown where statistically significant differences were found between transgenic and control groups. (**B**) The number of anti-NP IgG1-secreting splenocytes was determined using an ELISPOT assay. Symbols represent the population mean at each dilution and error bars show the standard error of the mean for each group. Statistical significance was found between control mice (n = 6) and B-B7hTg mice (n = 5), but not DC-B7hTg mice (n = 6). (**C**) The number of B220^+^IgD^neg-^GL7^+^Fas^+^ germinal center B cells was determined by flow cytometry. Each symbol represents one mouse and bars indicate the mean of each group. The data are representative of at least 3 independent experiments.

We observed increased production of anti-NP IgG1 antibodies (as well as other IgG isotypes not shown) from overnight cultures of splenocytes isolated from B-B7hTg mice on the B7h^+/+^ background 7 days after immunization, compared with wild-type controls (Figure 4A). To determine whether this phenotype resulted from increased plasma cell numbers or from increased antibody production per plasma cell, we measured the number of NP-specific IgG1-secreting splenocytes by ELISPOT analysis. Increased numbers of anti-NP antibody-producing cells were observed in splenocytes from B-B7hTg mice, but not from DC-B7hTg mice, when compared to wild-type controls (Figure 4B). Increased plasma cell numbers in immunized B-B7hTg mice did not result from an increase in the number of germinal center B cells that can give rise to antibody-secreting plasma cells [31], as equivalent numbers of germinal center B cells were detected by flow cytometry at the peak of the germinal center response in spleens from both immunized B-B7hTg and DC-B7hTg mice on the B7h^+/+^ background, in comparison to wild-type controls (Figure 4C). These results suggest that the number of plasma cells generated in response to T cell-dependent antigens is controlled in part by the level of ICOS costimulation provided by plasma cells, as transgenic B7h overexpression on this cell type resulted in an increase in both the number of responding plasma B cells and the serum concentration of antigen-specific IgG antibodies. 

### **B7h expression on plasma B cells regulates high affinity antibody production**

**Figure 5:**
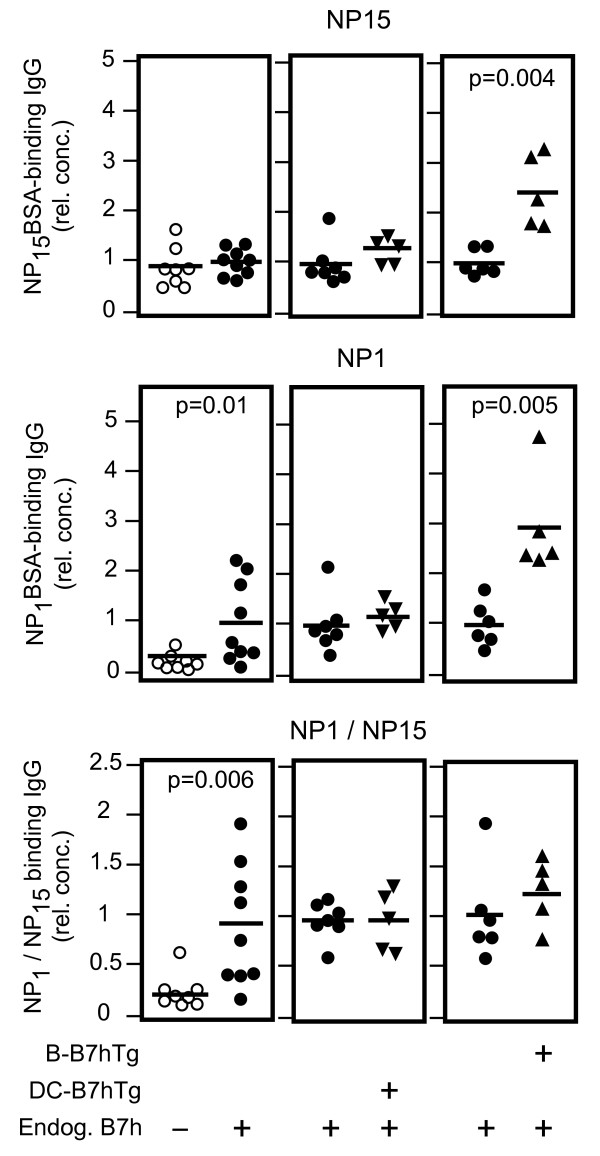
**The level of B7h on plasma B cells, but not on dendritic cells, regulates high-affinity antibody responses to T cell-dependent antigens.** Transgenic B-B7hTg and DC-B7hTg mice on the B7h^+/+^ background, and non-transgenic B7h^+/+^ and B7h^−/−^ controls were immunized with NP-CGG in alum, and the affinity of serum anti-NP IgG responses were assessed by ELISA after 21 days. Total NP-specific IgG (captured with NP_15_BSA; upper panels), high-affinity anti-NP IgG (captured with NP_1_BSA; middle), and the ratio of high affinity/total NP-specific IgG (lower panels) are shown relative to wild-type responses. Each symbol represents an individual mouse and bars indicate the mean of each group. The data were normalized such that the mean of the wild-type group was equal to 1. P values are shown for comparisons where differences were statistically significant. The data are representative of three independent experiments.

The primary immune response to NP-CGG immunization leads to production of low affinity antibody-secreting plasma cells early in the response (peaking at day 7), while the germinal center reaction selects for plasma cells secreting mutated high-affinity antibodies at later time points [32,33]. Having observed an increase in total antigen-specific IgG production in immunized B-B7hTg mice on the B7h^+/+^ background over non-transgenic controls on day 7, we wondered whether increased B7h expression on plasma B cells in these mice also affected the production of high affinity IgG. We therefore investigated the affinity maturation of anti-NP IgG responses in transgenic and control mice by examining the ratio of high affinity anti-NP IgG (IgG binding NP_1_BSA) to total anti-NP IgG (IgG binding NP_15_BSA) by ELISA in sera obtained 21 days after immunization (Figure 5). In comparison to wild-type B7h^+/+^ controls, high-affinity NP-specific IgG responses were defective in B7h^−/−^mice, consistent with previous observations [7]. In contrast, both total and high affinity NP-specific IgG were increased in B-B7hTg mice on the B7h^+/+^ background, but not in DC-B7hTg mice, when compared with wild-type B7h^+/+^ controls. Thus, enhanced B7h expression on plasma B cells, but not dendritic cells, markedly increased the production of both total and high affinity anti-NP IgG antibodies after immunization. 

### **B7h expression on dendritic cells selectively regulates IgG2a isotype usage**

In contrast to our observations in B-B7hTg mice, increased B7h expression on dendritic cells in DC-B7hTg mice did not affect the magnitude of IgG responses. Parallel immunization experiments with DC-B7hTg mice on the B7h^+/+^ background and non-transgenic littermate controls revealed no significant differences in either total IgG or IgM anti-NP responses (Figure 3B). Although overall IgG responses were unaffected, a selective increase in anti-NP IgG2a production was observed in DC-B7hTg mice when compared with wild-type controls, indicating a specific role for ICOS signaling mediated by dendritic cells in promoting antibody isotype switching to IgG2a. In agreement with these results, the IgG2a/IgG1 ratio of NP-specific serum antibodies was also increased in DC-B7hTg mice on the B7h^+/+^ background compared with wild-type control mice 14 days after immunization (Figure 3C).

### **B7h expression on dendritic cells contributes to germinal center formation**

**Figure 6:**
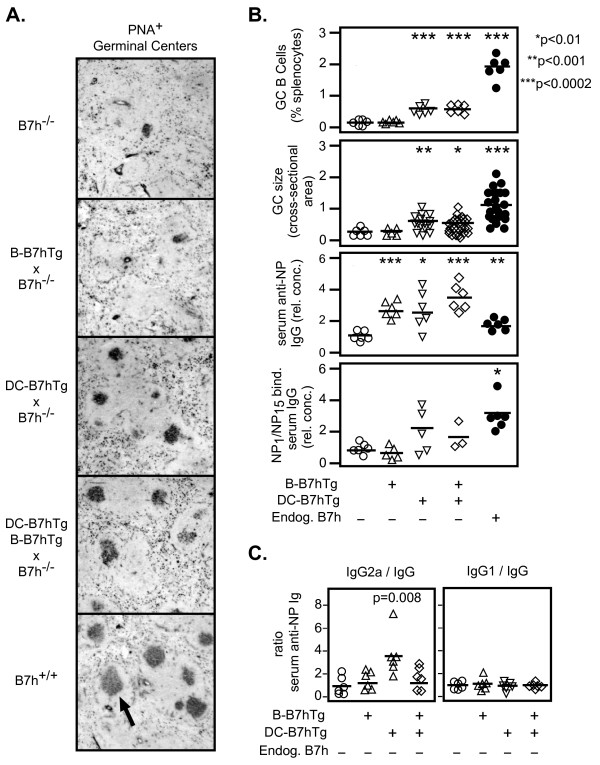
**B7h expression on DCs, but not plasma B cells, contributes to germinal center formation and enhances IgG2a isotype usage.** Transgenic mice on the B7h^−/−^ background were immunized with NP-CGG in alum, and analyzed after 14–21 days. (**A**) Splenic cryosections were analyzed by immunohistochemistry on day 14 to reveal PNA+ germinal centers (indicated by arrow). Images are representative of larger fields shown in Additional file 2: Figure S2. Original total magnification was 64X. (**B**) The number of germinal center B cells measured by flow cytometry, the relative cross-sectional area of germinal centers determined by imaging, and serum anti-NP IgG responses measured by ELISA at day 14 are shown. The relative affinities of serum anti-NP antibody responses were measured by the ratio of NP_1_/NP_15_-binding serum IgG on day 21. (**C**) The ratios of NP-specific IgG2a and IgG1 to total NP-specific IgG in serum on day 14, determined by ELISA, are shown. For ELISA and flow cytometry data, each symbol represents an individual immunized mouse. For GC size analysis, each symbol represents an individual germinal center. The mean of each group is indicated by a solid bar, and p values are shown where significance was detected (Student’s *t* test, p < 0.05) between the B7h^−/−^ group and transgenic or B7h^+/+^ groups. For ELISA data, the mean values from control B7h^−/−^ groups were set to a value of 1. The data are representative of three independent experiments.

Studies of knockout mice have demonstrated the requirement of ICOS signaling for germinal center formation [13-15]. However, the identity of the B7h-expressing cell type(s) that lead to germinal center formation through ligation of ICOS on activated T cells have not been fully defined. To determine whether expression of B7h on DCs or plasma B cells contribute to germinal center formation, we crossed DC-B7hTg and B-B7hTg mice with B7h^−/−^ mice, and examined germinal center activity both by immunohistochemical analysis of frozen spleen sections and by flow cytometry 14 days after immunization (Figures 6A, 6B, and Additional file 2: Figure S2). Consistent with prior studies of knockout mice [13-15], PNA+ germinal centers were small and rare in immunized B7h^−/−^ mice, but large and numerous in immunized B7h^+/+^ controls. Restoration of B7h expression on dendritic cells (DC-B7hTg), but not on plasma B cells (B-B7hTg), partially rescued germinal center formation in B7h^−/−^ mice, leading to increases in both the size of germinal centers and the number of germinal center B cells, suggesting that ICOS signaling in T cells mediated by contact with B7h-expressing DCs, but not plasma B cells, contributes to germinal center formation. 

Both the total amount of antibody produced and the affinity of the observed antibody responses, as reflected by the ratio of NP_1_BSA-binding/NP_15_BSA-binding serum IgG at day 21, were dramatically reduced in B7h^−/−^ mice compared with B7h^+/+^ controls (Figure 6B). While B7h expression on either plasma B cells or DCs in mice on the B7h^−/−^ background was sufficient to restore total IgG responses to wild-type levels, the affinity of the antibody responses in these mice did not reach the level observed in B7h^+/+^ mice. However, partial rescue of germinal center formation in B7h^−/−^ mice by expression of B7h on DCs (DC-B7hTg), but not on plasma B cells (B-B7hTg), was correlated with an increase in the affinity of the antibody response over B7h^−/−^ controls, suggesting that augmentation of germinal center formation by DC-mediated ICOS costimulation lead to increased antibody affinity maturation.

To confirm our observations in transgenic mice on the B7h^+/+^ background, we also assessed antibody isotype usage in transgenic mice on the B7h^−/−^ background by ELISA 14 days after immunization. B7h expression on DCs in DC-B7hTg mice on the B7h^−/−^ background again selectively increased isotype switching to IgG2a, reflected by an increase in the NP-specific IgG2a/IgG ratio, but not the IgG1/IgG ratio, over B7h^−/−^ controls (Figure 6C). In contrast, antibody isotype usage was equivalent between B-B7hTg mice on the B7h^−/−^ background and non-transgenic controls. Thus, our results suggest ICOS costimulation mediated by DCs, but not plasma B cells, contributes to germinal center formation and selectively enhances IgG2a isotype usage.

## Discussion

Analyses of both ICOS^−/−^ and B7h^−/−^ mice have demonstrated the critical requirement of ICOS costimulation for normal germinal center formation, antibody isotype switching, affinity maturation, and antibody production in T cell-dependent antibody responses [1,7,13,14]. However, knockout mice exhibit defects at multiple stages of T cell-dependent antibody responses, ICOS costimulation can have diverse effects on immune responses depending on the context of signaling, and B7h is expressed on multiple antigen presenting cell types involved in antibody responses. Thus, studies of knockout mice could not dissect the potential cell type-specific roles of B7h expression on antigen presenting cells in T cell-dependent antibody responses. We have extended previous studies using transgenic mice with lineage-restricted B7h expression to define selective roles for ICOS costimulation mediated by DCs and plasma B cells in the context of antibody responses.

Our results demonstrate that endogenous B7h expression is high on naïve and germinal center B cells, but is reduced on activated and plasma B cells, and transgenic induction of B7h expression on activated and plasma B cells in B-B7hTg mice enhances the number of antibody-secreting cells produced in response to T cell-dependent antigens. The observed expression pattern of the B-B7h transgene may most likely be explained as a result of the use of an immunoglobulin promoter in place of the endogenous B7h promoter sequences to drive transgene expression, as the immunoglobulin promoter is upregulated in plasma B cells, and possibly also by insertion of the transgene at a genomic location that is available for transcription only in activated B cells. The latter possibility could potentially be problematic if the transgene insertion disrupted an endogenous gene that is important for B cell function. Fortunately, we did not observe any defects in B cell function in B-B7hTg mice in vitro or in vivo, leading us to conclude that such a disruption is unlikely to have occurred in this case.

While we observed induction of B7h expression in B-B7hTg B cells after BCR cross-linking in vitro, we were unable to specifically identify analogous recently activated B cells in vivo in our system, as the frequency of NP-reactive B cells in naïve mice is very low, and the low affinity of primary naïve B cells for NP precluded direct identification of NP-specific cells by FACS based on NP binding in the first few days after immunization in our hands. Although we could not directly measure B7h expression levels on recently activated B cells in vivo, our in vitro results suggest that the B-B7h transgene may lead to overexpression of B7h on these cells in the first 2–3 days, a time frame in which they interact with cognate activated T cells in interfolicular regions [34,35]. Thus, it is possible that increased ICOS costimulation in T cells interacting with responding follicular B cells in the first 2–3 days after immunization contributed to the increased antibody responses in B-B7hTg mice. However, it is not clear how ICOS signaling in early cognate B-T interactions could augment the levels of both pre-germinal center low affinity and post-germinal center high affinity antibody production without affecting the number of germinal center B cells.

In addition to expression on in vitro-activated B cells, the B-B7h transgene consistently led to dramatic overexpression of B7h on responding plasma B cells in vivo, and an expansion of these cells in comparison to non-transgenic control mice. The correlation between B7h overexpression on plasma cells and cellular expansion of plasma cells in B-B7hTg mice suggests that differentiated plasma B cells make contact with ICOS-expressing activated T cells, and this interaction regulates the magnitude of antibody responses based in part on the level of B7h expression on B cells, which influences the strength of ICOS signaling in cognate T cells. This putative plasma cell-T cell interaction could potentially occur between Tfh cells and newly differentiated plasma cells in germinal centers, and/or between activated Th cells and plasma cells that co-localize in peri-arteriolar lymphoid sheaths (PALS) in lymph nodes and spleen [24]. The observed increase in the number of antibody-secreting cells in B-B7hTg mice at day 7 implies that plasma cell-T cell interactions that regulate antibody responses can likely occur outside of germinal centers, as the majority of antibody secreting cells at this early time point are not germinal-center derived [33]. 

ICOS costimulation in T cells is dynamically regulated on multiple levels. A previous study generated transgenic mice that overexpress ICOS on all T cells [36], and made the counterintuitive finding that antibody and germinal center responses were reduced in ICOS transgenic mice, as a result of the increased clipping of B7h from the cell surface of APCs after contact with ICOS [27]. The resulting reduction of B7h expression on APCs in ICOS transgenic mice caused a reduction of ICOS costimulation in vivo compared with WT mice, leading to defects in B cell responses to T cell-dependent antigens. In addition to ICOS contact, previous in vitro studies have shown that IL4-R and antigen receptor signals reduce cell surface B7h levels on B cells through transcriptional and post-transcriptional mechanisms, while the addition of CD40, TLR7/8 and/or TLR9 signals can limit the extent of B7h downregulation after activation or ICOS contact, causing a relative increase in ICOS co-stimulation in cognate T cells [26,27]. In our B-B7hTg model the B7h transgene was induced on activated B cells as a result of the transgenic promoter, leading to an increase in cell surface B7h levels on activated B cells, which was most notable in vivo on differentiated plasma B cells. Ultimately this increased cell surface B7h expression led to expansion of plasma cells in vivo, presumably through increased ICOS co-stimulation in cognate T cells.

These results suggest that B cells regulate the magnitude of antibody responses in part by modulating B7h levels in response to multiple signaling pathways. In this model, physiological settings where endogenous B7h expression on B cells is enhanced or stabilized during antibody responses, such as by CD40 or TLR9 signaling, are predicted to lead to increased class-switched antibody production. Conversely, the results of studies of ICOS transgenic mice suggest that stimuli that lead to an increase in ICOS expression on T cells may counter-intuitively reduce antibody responses through increased clipping of B7h from the cell surface of cognate APCs.

Our results have potential practical implications for the improvement of vaccination strategies, as they demonstrate that ICOS signaling is not only required for many aspects of T cell-dependent antibody responses, but that the strength of ICOS signaling, which is controlled in part by changing levels of B7h on APCs, can modulate antibody responses in the right context. Hence, vaccination strategies that increase ICOS costimulation either directly with ICOS agonists, or indirectly by attempting to stabilize B7h expression on B cells through CD40 or TLR signaling, may be useful to augment the magnitude of vaccine-induced high affinity antibody responses. Conversely, ICOS blockade may be a useful strategy for reducing the expansion of plasma cells secreting pathogenic antibodies in the setting of B cell-mediated autoimmunity [37,38]. Interactions between plasma B cells and ICOS+ T cells in the kidneys of lupus patients have been correlated with exaggerated plasma cell generation and pathogenic antibody production [39]. Our data suggest that the level of ICOS signaling in such interactions can regulate the plasma cell response, and blocking ICOS costimulation in this setting may be a useful therapeutic strategy to reduce the production of auto-antibodies.

Our results show that overexpression of B7h on activated and plasma B cells led to an increase in high-affinity IgG production 21 days after immunization (Figure 5). In this experiment, although a statistically significant difference in high-affinity NP_1_-binding IgG could be detected between B7h^+/+^ and B7h^−/−^ mice, the difference in total NP_15_-binding IgG was not significant. This was surprising, as numerous studies have reported defects in class-switched antibody production in B7h^−/−^ and ICOS^−/−^ mice or after ICOS blockade in WT mice [7,14,15,40]. However, several studies showed a decrease in IgG1, but not IgG2a production, after primary immunization [7,40], suggesting that all isotypes may not be equivalently affected, and the extent of the observed differences vary between studies using different immunogens, adjuvants, and timing of sample collection [14]. In the case of the experiment shown in Figure 5, we may have failed to detect a difference between B7h^+/+^ and B7h^−/−^ responses because we measured all IgG isotypes together, and took blood samples at day 21 for the purpose of measuring affinity maturation, in contrast to previous reports that show differences specifically for the IgG1 isotype, at 7 and 14 days after immunization. In fact, in a similar experiment measuring total NP_15_-binding IgG at day 14, we did detect a statistically significant difference between B7h^+/+^ and B7h^−/−^ animals (Figure 6B), suggesting that while IgG responses in B7h^−/−^ mice are initially deficient, at later time points total antigen-specific IgG in immunized B7h^−/−^ mice may reach wild-type levels, although high-affinity (NP_1_-binding) antibody responses were deficient as a result of limited germinal center activity in knockout mice.

Initial studies reported Th1 polarization [16], reduced IL-4 production [15,41], and inhibition of Th2-mediated lung mucosal inflammation [40,42] in the absence of ICOS signaling, suggesting a role for ICOS in Th2 polarization. Additional studies have paradoxically shown amelioration of Th1-mediated allograft rejection [43], EAE [25,44], and diabetes [45] in the absence of ICOS signaling, as well as enhancement of Th1-driven IgG2a production by an activatory B7h-Ig fusion protein [9]. Our results suggest these observations are not contradictory, but rather the outcome of ICOS signaling in activated T cells is highly dependent on the context of B7h-ICOS interactions, as B7h expressed on dendritic cells, but not plasma B cells, can specifically enhance Th1-driven IgG2a responses, while B7h expression on plasma B cells increases IgG production of all isotypes. This may in part reflect the fact that responding DCs and plasma B cells can localize to separate micro-environments, and potentially interact with CD4+ T cells in different states of differentiation that regulate distinct aspects of antibody responses.

In addition to the effect on antibody isotype switching, we show that B7h expression on dendritic cells also contributes to germinal center formation, which leads to antibody affinity maturation. However, the number of responding germinal center B cells in mice that only express B7h on DCs (DC-B7hTg x B7h^−/−^) was reduced in comparison to wild-type B7h^+/+^ mice (Figure 5b), suggesting that additional ICOS costimulation mediated by other cell types is necessary for optimal germinal center responses and antibody affinity maturation. Since ICOS expression is high on Tfh cells in germinal centers and endogenous B7h expression is high on germinal center B cells, it is likely that germinal center B cell-mediated ICOS signaling in Tfh cells also contributes to the size and dynamics of germinal centers and the extent of antibody affinity maturation in wild-type mice.

## Conclusions

In summary, ICOS costimulation mediated by plasma B cells and dendritic cells have distinct functions in T cell-dependent antibody responses. While ICOS signaling in T cells mediated by B7h-expressing DCs contributes to germinal center formation and can affect antibody isotype usage, the B7h expression level on plasma B cells regulates the overall magnitude of antibody responses to T cell-dependent antigens without affecting antibody isotype usage or the number of germinal center B cells. The latter result suggests a control point that may be useful for enhancement of vaccine-induced antibody responses, or reduction of pathogenic antibody responses, through modulation of ICOS signaling.

## Methods

### **Transgenic mice**

All work involving animals was performed in accordance with the NIH Office of Laboratory Animal Welfare guidelines and was approved by the UC Berkeley Institutional Animal Care and Use Committee.

The construct driving B7h expression in B-B7hTg mice was created by modifying an existing vector that utilizes an immunoglobulin V_H_ promoter and enhancer for cDNA expression in B cells [29], in which transcription is driven by a 0.9 kb XbaI fragment containing the intronic Eμ enhancer and a 1.4 kb fragment of the V_H_17.2.25 promoter ending at a SalI cloning site at position +38, upstream of the Kozak ATG sequence at +47 [46]. A SalI-NotI blunted B7h-GFP cDNA insert, encoding for a previously described B7h-GFP fusion protein [5], where the N terminus of GFP was fused to the C terminal end of the cytoplasmic tail of full-length B7h, was sub-cloned into the SalI site of the vector, which was additionally modified to replace the SV40 splice and polyA sequences with a BamHI-NotI insert containing the human growth hormone (hGH) minigene from the p1017-lck expression vector plasmid [47]. A 6.9 kb NotI insert lacking plasmid vector sequences was microinjected into (CBA X B6)F_1_ embryos. The B-B7hTg line utilized in these studies was subsequently backcrossed to B6 mice for at least 10 generations.

The construct driving B7h expression in DC-B7hTg mice was created by modifying an existing transgenic vector designed for cDNA expression in DCs driven by the CD11c promoter upstream of the rat β-globin splice site and polyA signal sequence [28]. A blunted 2.5 kb B7h-IRES-GFP cDNA insert, excised from an MSCV-IRES-GFP retroviral vector [27,48] expressing the B7h cDNA [5], was then sub-cloned into a PmeI site that was introduced into the EcoRI cloning site of the vector by annealing overlapping primers 5— AATTGTTTAAAGCCGG-3′ and 5′-ATTCCGGGTTTAAAC-3′ to the EcoRI-digested vector. A 9 kb NotI-XhoI insert lacking plasmid vector sequences was then microinjected into B6 embryos. The DC-B7hTg line reported here was selected for high B7h expression on CD11c^+^ cells. Transgenic lines were crossed onto B7h^−/−^ mice on the C57Bl6/J background, a generous gift of Dr. Tak Mak [7], and were maintained on the C57Bl6/J background.

### **Immunization**

NP-OsU (Biosearch Technologies) was conjugated to chicken gamma globulin (CGG; Jackson Immunoresearch) or bovine serum albumin (BSA; Sigma) to form NP-CGG or NP-BSA at different conjugation ratios that were confirmed by spectroscopy. Age-matched groups of mice were immunized intraperitoneally in the lower right quadrant with 100 μg NP_10_CGG precipitated in alum, in a final volume of 100 μL phosphate-buffered saline (PBS). Levels of NP-specific antibodies in sera were detected using ELISA plates (Costar) coated with 5 μg/mL NP_15_BSA or NP_1_BSA in PBS overnight at 4°C, and isotype-specific HRP-conjugated anti-mouse IgG or IgG1 (Southern Biotech), or biotinylated anti-mouse IgG2a, IgG2b, IgG3 or IgM (Pharmingen) detection reagents. Plates were developed with streptavidin-conjugated HRP and ABTS substrate (Pharmingen), and absorbance at 405 nm of a two-fold dilution series in the linear range was used to calculate relative amounts of antiNP antibodies. ELISPOT analysis of antibody producing cells was conducted using MultiScreen-IP plates (Millipore) coated with 50 μg/mL NP_15_BSA. Purified splenocytes were serially diluted in duplicate onto blocked washed plates in lymphocyte media and cultured overnight at 37°C. NP-specific spots were detected with an HRP-conjugated goat anti-mouse IgG1 antibody (Southern Biotech), developed using the AEC chromagen kit (Sigma), and ELISPOTs were counted using a dissecting microscope.

### **Flow cytometry and cells**

Immature dendritic cells were derived by isolating bone marrow from the long bones of the legs and culturing in IMDM containing 10% FBS, 1% Pen/Strep, and 500 ng/mL GM-CSF (R&D Systems) for 7 days. Splenic dendritic cells were enriched in some experiments by injecting mice subcutaneously with an FLT3L-secreting B16 tumor cell line 14–21 days before analysis. Dissected spleens were injected with 400 U/mL collagenase in PBS to disaggregate splenocytes, and dead cells were removed by density gradient centrifugation prior to isolation of DCs with anti-CD11c microspheres (Miltenyi Biotec). Dendridic cells were activated in some experiments by culture for 16 hours in the presence of 1 μg/mL LPS (Sigma). B and T cells were purified from mice as described previously [26] and activated by culture in 96-well plates at 10 X 10^6^ cells/mL for 16–72 hours in the presence of 5 μg/mL goat anti-mouse-IgM F(ab’)_2_ (Jackson Immunoresearch), 250 nm PMA (Sigma) and 2 μM Ionomycin (Sigma), 10 μg/mL LPS (Sigma), 5 μg/mL CpG oligonucleotide 1826 (5′-TCCATGACGTTCCTGACGTT-3′), or 5 ng/mL IL-4 (R&D Systems).

Splenocyte suspensions or purified cells were stained for flow cytometric analysis as described [27] using the following reagents: IgD-FITC (eBioscience), B220-PETexasRed (Pharmingen), Fas-PECy7 (Pharmingen), syndecan-1-APC (Pharmingen), CDllc-PE (Caltag), CD11c-PECy7 (Pharmingen), B7.2-PE (Pharmingen), purified rat IgM anti-GL7 (eBioscience) followed by anti-rat IgM-PE secondary (eBioscience), and 22D7 hamster anti-mouse B7h [13] followed by streptavidin-PE (eBioscience) or streptavidin-PECy5 (Pharmingen). For cell surface B7h analysis, splenocytes were cultured for 2 hours at 37°C in lymphocyte media and blocked with 220 μg/mL Syrian hamster gamma globulin (Jackson ImmunoResearch). Germinal center B cells were gated as B220^+^IgD^neg^GL7^+^Fas^hi^, plasma cells were IgD^neg^Syndecan^+^B220^lo^, naïve B cells were B220^+^IgD^+^, and dendritic cells were CD19^neg^CD11c^+^.

### **Immunohistochemistry**

Spleens from immunized mice were snap frozen in OCT, and glass slides with microtome sliced issues sections cut to 5–7 μm were stained with biotinylated peanut agglutinin (PNA) in PBS. PNA+ germinal centers were revealed using an ABC Elite staining kit with DAB substrate (Vector labs). Slides were imaged on a Zeiss NeoLumar stereomicroscope using a 0.8x, 80 mm objective at 64X total magnification. Images were acquired with a MicroPublisher 3.3 Qimaging camera and QImaging software; contrast of final images was adjusted uniformly to all images using Adobe Photoshop.

### **Statistical analysis**

Statistical significance between groups of mice was evaluated using Student’s *t* test (one-tailed, two sample, unequal variance). All analyses returning a p value < 0.05 are labeled. Analyses where no p value is shown returned p > 0.05.

## Competing interests

The authors declare that they have no competing interests.

## Authors’ contributions

SB generated the DNA construct used to create DC-B7hTg mice. LL generated and initially characterized the B-B7hTg mouse line*.* KL generated the DC-B7hTg mouse line, performed all experiments with results shown, and drafted the manuscript. BS contributed to the study design and supervised the studies. All authors read and approved the final manuscript.

## Supplementary Material

Additional file 1 **Figure S1.** Normal lymphocyte populations in resting B7hTg mice. Splenic lymphocyte populations were assessed in resting B-B7hTg and DC-B7hTg animals on the B7h^+/+^ background by flow cytometry. The number of lineage marker positive cells is plotted as a percentage of total splenocytes, with representative gating shown on the left. Each symbol represents an individual animal, with the mean of each group represented by a solid bar. Click here for file

Additional file 2 **Figure S2.** Defective germinal center formation is restored by expression of B7h on CD11c+ dendritic cells, but not on plasma B cells. Splenic cryosections from immunized mice at day 14 were analyzed by immunohistochemistry to detect PNA+ germinal centers. Images correspond to data presented in Figure 5. Original magnification was 64x. Click here for file
