# Supplementary material for: B7h-expressing dendritic cells and plasma B cells mediate distinct outcomes of ICOS costimulation in T cell-dependent antibody responses
Source: BMC Immunol. 2012 Jun 11;13:29. doi: 10.1186/1471-2172-13-29 (PMC3477010; doi:10.1186/1471-2172-13-29)
Supplement: Additional file 1 — Figure S1. Normal lymphocyte populations in resting B7hTg mice. Splenic lymphocyte populations were assessed in resting B-B7hTg and DC-B7hTg animals on the B7h+/+ background by flow cytometry. The number of lineage marker positive cells is plotted as a percentage of total splenocytes, with representative gating shown on the left. Each symbol represents an individual animal, with the mean of each group represented by a solid bar. [file 1471-2172-13-29-S1.pdf]

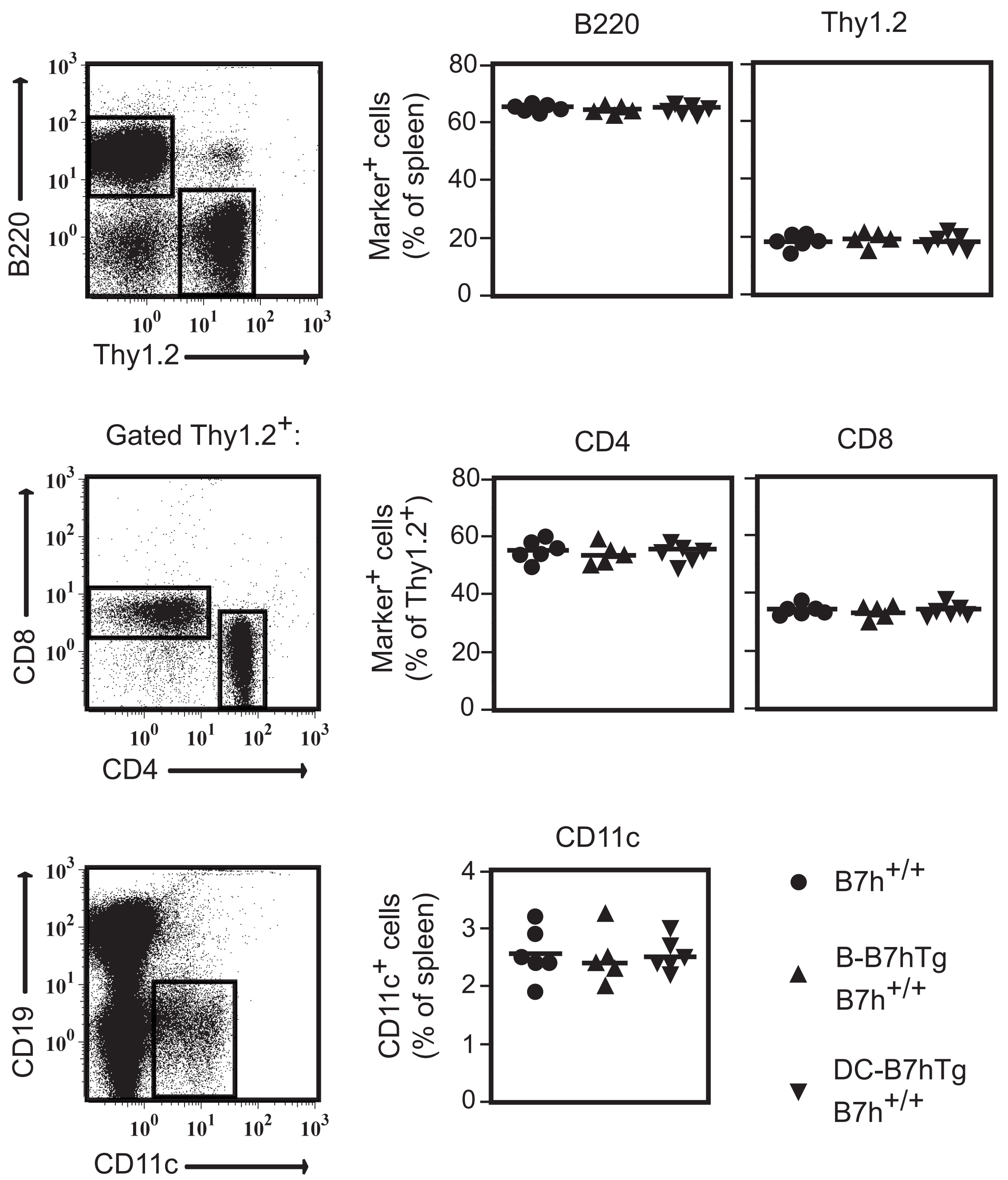

Figure S1. Normal lymphocyte populations in resting B7hTg mice. Splenic lymphocyte populations were assessed in resting B-B7hTg and DC-B7hTg animals on the B7h$^{+/+}$ background by flow cytometry. The number of lineage marker positive cells is plotted as a percentage of total splenocytes, with representative gating shown on the left. Each symbol represents an individual animal, with the mean of each group represented by a solid bar.
