# Supplementary material for: B7h-expressing dendritic cells and plasma B cells mediate distinct outcomes of ICOS costimulation in T cell-dependent antibody responses
Source: BMC Immunol. 2012 Jun 11;13:29. doi: 10.1186/1471-2172-13-29 (PMC3477010; doi:10.1186/1471-2172-13-29)
Supplement: Additional file 2 — Figure S2. Defective germinal center formation is restored by expression of B7h on CD11c+ dendritic cells, but not on plasma B cells. Splenic cryosections from immunized mice at day 14 were analyzed by immunohistochemistry to detect PNA+ germinal centers. Images correspond to data presented in Figure 5. Original magnification was 64x. [file 1471-2172-13-29-S2.pdf]

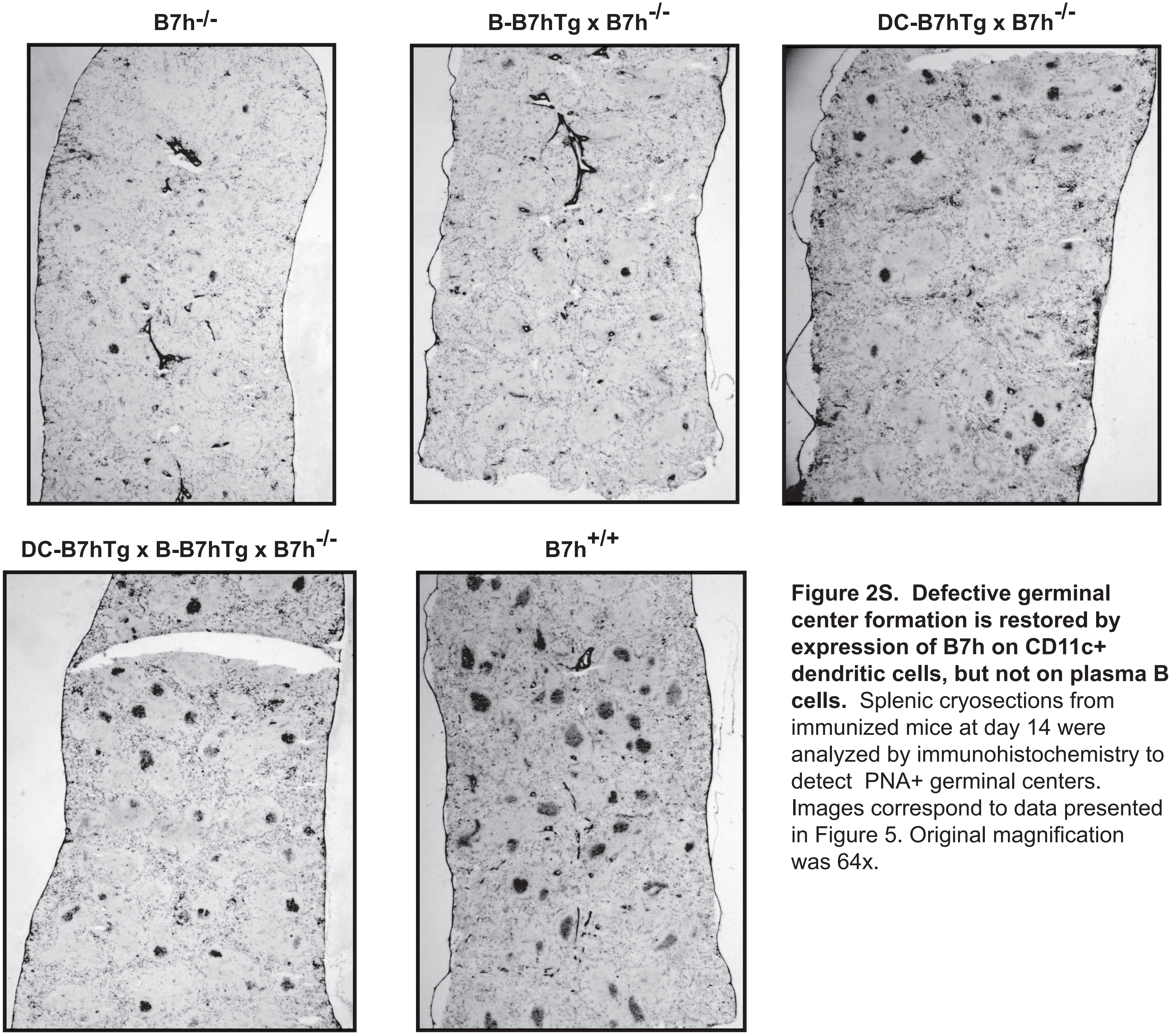

Figure 2S. Defective germinal center formation is restored by expression of B7h on CD11c+ dendritic cells, but not on plasma B cells. Splenic cryosections from immunized mice at day 14 were analyzed by immunohistochemistry to detect PNA+ germinal centers. Images correspond to data presented in Figure 5. Original magnification was 64x.
